# Endoplasmic Reticulum Stress in Heat- and Shake-Induced Injury in the Rat Small Intestine

**DOI:** 10.1371/journal.pone.0143922

**Published:** 2015-12-04

**Authors:** Peng Yin, Jianqin Xu, Shasha He, Fenghua Liu, Jie Yin, Changrong Wan, Chen mei, Yulong Yin, Xiaolong Xu, Zhaofei Xia

**Affiliations:** 1 CAU-BUA TCVM Teaching and Researching Team, College of Veterinary Medicine, China Agricultural University (CAU), Beijing, PR China; 2 College of Animal Science and Technology, Beijing University of Agriculture (BUA), Beijing, PR China; 3 Key Laboratory of Agro-ecological Processes in Subtropical Region, Institute of Subtropical Agriculture, Chinese Academy of Sciences, Changsha, 410125, Hunan, China; University Hospital Carl Gustav Carus Dresden, GERMANY

## Abstract

We investigated the mechanisms underlying damage to rat small intestine in heat- and shake-induced stress. Eighteen Sprague-Dawley rats were randomly divided into a control group and a 3-day stressed group treated 2 h daily for 3 days on a rotary platform at 35°C and 60 r/min. Hematoxylin and eosin-stained paraffin sections of the jejunum following stress revealed shedding of the villus tip epithelial cells and lamina propria exposure. Apoptosis increased at the villus tip and extended to the basement membrane. Photomicrographs revealed that the microvilli were shorter and sparser; the nuclear envelope invaginated and gaps in the karyolemma increased; and the endoplasmic reticulum (ER) swelled significantly. Gene microarray analysis assessed 93 differentially expressed genes associated with apoptosis, ER stress, and autophagy. Relevant genes were compiled from the Gene Ontology (GO) and Kyoto Encyclopedia of Genes and Genomes (KEGG) databases. Forty-one genes were involved in the regulation of apoptosis, fifteen were related to autophagy, and eleven responded to ER stress. According to KEGG, the apoptosis pathways, mitogen-activated protein kinase(MAPK) signaling pathway, the mammalian target of rapamycin (mTOR) signaling pathway, and regulation of autophagy were involved. Caspase3 (Casp3), caspase12 (Casp12), and microtubule-associate proteins 1 light chain 3(LC3) increased significantly at the villus tip while mTOR decreased; phosphorylated-AKT (P-AKT) decreased. ER stress was involved and induced autophagy and apoptosis in rat intestinal damage following heat and shake stress. Bioinformatic analysis will help determine the underlying mechanisms in stress-induced damage in the small intestine.

## Introduction

Severe physical stress can cause gastrointestinal (GI) dysfunction and pathology, including stress ulcers, multiple organ dysfunction, and increased intestinal permeability [1]. High temperature and shaking, as two important stimuli, have significant effects on humans and animals, especially in summer. Studies have reported that peripheral blood flow increases to dissipate internal body heat, resulting in a significant reduction in blood flow to the small intestine during heat stress [2]. This results in intestinal mucosal barrier dysfunction and induced ischemia at the villus tip [3–5]. Ischemia of the small intestine has been found to promote formation of reactive oxygen species [6] and high levels of free radicals lead to oxygen radical damage at the intestinal mucosa [7]. Heat stress-related oxidative stress causes apoptosis in the rat small intestine [8], also seen with simultaneous heat and shaking in rats [9–11].

The process of protein folding is particularly sensitive to stress, either endogenous or exogenous. The accumulation of unfolded proteins in the ER causes ER stress and induces the unfolded protein response (UPR), which alleviates stress by up-regulating protein folding and degradation pathways in the ER inhibiting protein synthesis [12–14]. Ischemia of the intestinal villus leads to oxidative stress and insufficient exogenous blood supply, resulting in limited nutrient delivery, and could induce further ER stress. Activation of the UPR on exposure to oxidative stress is an adaptive mechanism to preserve cell function and survival. Calcium and free radicals are essential mediators linking ER stress to metabolic processes [15] and caspase 12 (Casp12) is thought to be a key mediator of ER stress-induced apoptosis [16, 17]. Also, ER stress inhibits serine/threonine protein kinase (AKT) phosphorylation through the up-regulation of telomere repeat binding factor 3 (TRB3), and induces apoptosis through a mechanism requiring phosphatidylinositol 3-kinase (PI3K)/AKT pathway [18].

ER stress stimulates the assembly of pre-autophagosomal structures [19]. The autophagy system is activated as a cell survival signaling pathway in response to ER stress [20]; however, if these mechanisms do not remedy the stress situation, persistent oxidative stress and protein misfolding initiate apoptotic cascades, presumably to eliminate unhealthy cells [13, 21, 22]. Autophagy and apoptosis are two distinct processes that play seemingly opposite biological roles in response to stress [23]. If protein aggregation is persistent and the stress cannot be resolved, signaling switches from pro-survival to pro-apoptotic [21]. In this case, UPR and ER stress may play critical roles in determining cell survival or cell death, which could reveal the underlying injury mechanisms induced by heat and shake stress.

Autophagy is a type of stress response [19, 24]. It provides the necessary amino acids [25, 26], eliminates a specific species of misfolded procollagen, and plays a protective role in cell survival in ER stress [27]. An accumulation of autophagosomes could reflect induction of autophagy [28]. mTOR adaptors were required in leucine-mediated autophagy inhibition [29] and ER stress negatively regulates the AKT/ tuberous sclerosis (TSC)/mTOR pathway to enhance autophagy [30]. If we hope to use autophagy to relieve intestinal damage caused by stress, it will be crucial to understand the details of its regulatory processes.

We questioned how autophagy and apoptosis genes functionally interact in ER stress, and how they are regulated to achieve synergy and flexibility in response to the damage induced by diverse stresses in rat small intestine; however, these questions are quite complicated, and have not been well elucidated. Gene expression profile analysis could provide more information on the relevant mechanisms. We describe our findings from a systems biology approach to reveal potential mechanisms in heat- and shake stress-induced rat small intestine injury, especially on the third day after stress. (Our previous work showed that the rat intestine suffered most on the third day after heat and shake stress.) We also report the findings of a morphological study as part of this research.

## Materials and Methods

### Animals and Groups

Eighteen male Sprague-Dawley rats weighing 200 ± 20 g (Beijing Vital River Laboratory, Animal Technology Co., Beijing, P. R. China) were housed at 25°C and 60% relative humidity for 1 week under a 12-h light/dark (19:00–07:00 h) cycle. On the eighth day, the rats were randomly divided into the following three groups: control (C), 1-day stress (S1d), and 3day stress (S3d) groups. Our previous study concluded that the day 2 stress group findings were not significantly different from the day 3 stress group results. Six rats in each group were housed in plastic cages (400 mm × 300 mm × 180 mm) with a layer of aspen shavings and provided free access to food and water. Additional data from rats subjected to the treatment protocol described below have been previously published [9].

Animals from the source colony were tested and found to be free of a list of pathogens and adventitious agents on arrival; details may be found at [http://www.vitalriver.com.cn/en/index_en.html]. All procedures performed on the animals were approved by the Animal Care and Use Committee of China Agricultural University (CAU) (permit number: 20121209–1). We followed the guidelines of the CAU Animal Care and Use Committee in handling the experimental animals during this study. The abbreviations of groups were listed in Table 1.

**Table 1:** List of abbreviations.

Name	Description	Name	Description
S1d	1-day stress group	S3d	3 day stress group
C	Control group	ER	Endoplasmic reticulum
GI	Gastrointestinal	GO	Gene ontology
KEGG	Kyoto Encyclopedia of Genes and Genomes	TEM	Transmission electron microscopy
TUNEL	Terminal deoxynucleotidyl transferase dutp nick end labeling	UPR	Unfolded protein response
Gene Description			
AKT	Serine/threonine protein kinase	P-AKT	Phosphorylated-AKT
Atg4b	Autophagy related 4b	Atg5	Autophagy related 5
Atg7	Autophagy related 7	Atg10	Autophagy related 10
Arsa	Arylsulfatase A	Atf4	Activating transcription factor 4
Atf6	Activating transcription factor 6	Bcl2l	Bcl2-like 1
Casp3	Caspase3	Casp8	Caspase8
Casp9	Caspase9	Casp12	Caspase12
Col4a3bp	Collagen type IV alpha 3 binding protein	Ctsd	Cathepsin D
Dap	Death-associated protein	Ddit3	DNA-damage-inducible transcript 3
Eif2s	Eukaryotic translation initiation factor-2s	Fas	Fas cell surface death receptor
Herpud1	Homocysteine-inducible endoplasmic reticulum stress-inducible ubiquitin-like domain member 1	Lamp1	Lysosomal-associated membrane protein 1
Lcn2	Lipocalin 2	LC3	Microtubule-associate proteins 1 light chain 3
Map1lc3b	Microtubule-associated protein 1 light chain 3 beta	MAPK	Mitogen-activated protein kinase
Mmp9	Matrix metallopeptidase 9	mTOR	The mammalian target of rapamycin
Os9	Osteosarcoma amplified 9	PI3K	Phosphatidylinositol 3-kinase
Scamp5	Secretory carrier membrane protein 5	Slc2a4	Solute carrier family 2 member 4
Tm9sf1	Transmembrane 9 superfamily member 1	TRB3	Telomere repeat binding factor 3
TSC	Tuberous sclerosis	Wipi1	WD repeat domain phosphoinositide interacting 1
Zbtb16	Zinc finger and BTB domain containing 1		

### Treatment and Sampling

The conditions of the treatment were as follows: 35°C with a vibration of 0.1 × g (relative centrifugal force) from 09:00–11:00 daily for 1 or 3 days, Sd1 and Sd3 groups, respectively. Rats’ rectal temperature and body weight were recorded daily before and after stress. On the first and third days, rats from each group were anesthetized by ether inhalational anesthesia (diethylether, PR China), exsanguinated immediately after anesthesia, and then sacrificed. Sections of the jejunum were rapidly excised and preserved as follows: (1) 1-cm sections were fixed for 48 h in 10% buffered formalin phosphate for later embedding in paraffin; (2) 1 mm^3^ samples were fixed for 48 h in 4% glutaraldehyde in 0.1 M cacodylate buffer (pH 7.4) for electron microscopy; and (3) 3-cm sections were washed using physiological saline (0.9%), then minced and separated into four sample tubes, frozen in liquid nitrogen, and stored at −80°C for DNA microarray and molecular biology experiments. Because the jejunum was the most severely injured in the S3d group, DNA microarray analysis was performed on tissues from this group. Rats’ rectal temperature, body weight, and HSPs were also determined.

### Morphological Analysis

Formalin-fixed samples were embedded in paraffin and transversely sectioned (5 μm thickness) then stained with hematoxylin and eosin (Sigma, St. Louis, MO, USA) after deparaffinization and dehydration [31]. The jejunal microstructures were examined using a BH2 Olympus microscope (DP71, Olympus, Tokyo, Japan) and analyzed using the Olympus Image Analysis System (version 6.0, Olympus). Additional paraffin sections were prepared for terminal deoxynucleotidyl transferase dUTP nick end labeling (TUNEL) fluorescein assay, (Roche, version 16.0, Philadelphia, PA, USA) and observed under fluorescence microscopy using an excitation wavelength of 450–500 nm and detection from 515–65 nm (green) (DP71, Olympus). Samples fixed using glutaraldehyde were washed in the same buffer and fixed for 1 h in cold 1% osmium tetroxide in cacodylate buffer. After graded ethanol solution dehydration, samples were embedded in araldite (EPON812, Emicron, Shanghai, PR China) and ultra-thin sections were made and stained with saturated uranyl acetate in 50% ethanol and lead citrate, then examined using transmission electron microscopy (TEM, 1230, JEOL, Tokyo, Japan).

### Immunohistochemistry

Intestinal sections were incubated for 15 min in 3% hydrogen peroxide/methanol to stop endogenous peroxidase activity, and then rinsed for 20 min with phosphate- buffered saline solution (PBS). The sections were then incubated in 5% goat serum in PBS for 30 min (#KGSP04 Histostain-plus kit, KeyGen Biotech, Nanjing, China). The antibodies included Casp3, 1:800, Casp12, 1:100, and mTOR (1:100), (#9664, #2202, #2983, respectively, Cell Signaling Technology, Danvers, MA, USA), and LC3 (L8918, Sigma, 1:200). Antibodies were pre-diluted, stored in PBS containing bovine serum albumin and 0.05% sodium azide, then applied to sections and incubated for 2 h at 37°C in a moist chamber, and subsequently treated with a secondary antibody for 30 min. Immune complexes were detected using streptavidin-peroxidase (#KGSP04 Histostainplus kit, KeyGen Biotech) for 30 min at room temperature. After three washes in PBS, immunoreactivity was determined using 0.1% 3,3-diaminobenzidine and 0.02% hydrogen peroxide for 5 min DAB substrate kit, Vector Laboratories, Burlingame, CA, USA). Finally, sections were counterstained with hematoxylin after rinsing in distilled water.

### Western Blot Analysis

Protein was extracted from rat small intestine using a total protein extraction kit (#K3011010, Biochain, Hayward, CA, USA), and the concentration determined using a bicinchoninic acid protein assay (#23225, Pierce, Rockford, IL, USA). A 30-μg sample of total protein was electrophoresed for 120 min at 100 V, before being transferred onto nitrocellulose membranes (#88585, Pierce). Membranes were blocked overnight at 48°C in SuperBlock T20 blocking buffer (#37536, Pierce). AKT, p-AKT, and GAPDH (#4685, #4060, #5174, respectively, CST, MA, USA), and LC3 (L8918, Sigma) antibodies were added to the blocking buffer (diluted according to the instruction manual) and incubated for 2 h under agitation. Blots were washed in PBS/tween-20 (T20) for 5 min with shaking. Blots were incubated with the secondary antibody (926–32211, IRDye 800CW goat anti-rabbit IgG (H+L). Proteins were detected using the Odyssey Infrared Imaging System (Li-Cor Biosciences, Lincoln, NE, USA). Quantification of digitized images of western blot bands from three biological replicates was performed using ImageJ software (National Institutes of Health, New York, NY, USA).

### Total RNA Isolation and Reverse Transcription

Total RNA was isolated from the jejunum using a phenol and guanidine isothiocyanate-based TRIzol reagent (Invitrogen, Carlsbad, CA, USA) according to the manufacturer’s instructions. The concentration and purity were assessed by a spectrophotometer (SmartSpec plus, Bio-Rad Laboratories, Inc., Hercules, CA, USA) based on the OD260/OD280 ratio [32].

Total RNA was reverse transcribed as follows: 2.0 μg RNA isolated from each tissue sample was added to 25 μL reaction solution containing 2.0 μL oligo-dT18, 5.0 μL deoxyribonucleoside triphosphates (dNTPs), 1.0 μL RNase inhibitor, 1.0 μL Moloney murine leukemia virus (M-MLV) transcriptase, 5.0 μL M-MLV reverse transcriptase reaction buffer (Promega, Madison, WI, USA) and RNase-free water. The reverse-transcription procedure was performed according to the manufacturer’s instructions (Promega) as follows: 70°C for 5 min and 42°C for 1 h. The reverse transcriptase products (cDNA) were stored at −20°C.

### Gene mRNA Expression Analysis by Real-Time PCR

The gene expression levels were determined by real-time PCR (RT-PCR) analysis. Quantitative PCR analysis was carried out using the DNA Engine Mx3000P® (Stratagene, La Jolla, California, USA) fluorescence detection system against a double-stranded DNA-specific fluorescent dye (Stratagene, La Jolla, CA, USA) according to optimized PCR protocols. β-actin was amplified in parallel with the target genes and used as a normalization control [33, 34]. The protocol was as follows: 95°C for 3 min, followed by 40 cycles of 95°C for 10 s, 60°C for 20 s, and 72°C for 60 s. For the dissociation curve, we incubated the amplified products at 95°C for 1 min and lowered the temperature to 55°C at a rate of 0.2°C/s while continuously measuring the fluorescence levels. Expression levels were determined using the relative threshold cycle (CT) method as described by the manufacturer (Stratagene). Each gene was calculated by evaluating the expression of 2^−ΔΔCT^, where ΔΔCT is the result of the following: [CTgene − CTβ-actin] (stress) − [CTgene − CTβ-actin] (control). The cDNA of each sample was subjected to RT-PCR using the primer pairs listed in Table 2. The PCR reaction (20 μL) contained 10 μL of SYBR Green PCR mix (Invitrogen), 0.3 μL of reference dye, 1 μL of each primer (both 10 μmol/L), and 1 μL of cDNA template.

**Table 2:** Real-time PCR primer sequences.

Gene	Primer sequence 5’-3’	Product size (bp)	Genbank number
β-Actin	Forward: TTGTCCCTGTATGCCTCTGG	218	NM_031144
	Reverse: ATGTCACGCACGATTTCCC		
Caspase-12	Forward CACTGCTGATACAGATGAGG	119	NM_130422
	Reverse CCACTCTTGCCTACCTTCC		
Caspase-8	Forward TGTGCATACATACACTCAAGACACA	250	NM_022277
	Reverse GCAACCTCAATGTAATACTGAAACC		
Caspase-9	Forward GAGGGAAGCCCAAGCTGTTC	69	NM_031632
	Reverse GCCACCTCAAAGCCATGGT		
ATF-4	Forward CCGAGATGAGCTTCCTGA	217	NM_024403
	Reverse CTCCTTGCCGGTGTCTGA		
Lcn2	Forward GATGTTGTTATCCTTGAGGCCC	162	NM_130741
	Reverse CACTGACTACGACCAGTTTGCC		
Dap	Forward TTCATTCGGGCAAACCTTTAGT	87	NM_022526
	Reverse TGGAACCAAATCTAGGAAGGGA		
Zbtb16	Forward AGGCCTCAAAGTTTCTCCACTG	286	NM_001013181
	Reverse TACCTGTCCCAGGCCAGTATTT		
ATF6	Forward GAATGGCTGCTTAATTTGCTCC	218	NM_001107196
	Reverse AAGTCCATCTTCGGTGATGAGG		
Herpud1	Forward ATACTTGGCTGCCACTGCT	237	NM_053523
	Reverse GTCTCGGTTTATCTCATCATCTT		
mTOR	Forward GTCACAATGCAGCCAACAA	591	NM_019906
	Reverse AACAAACTCGTGCCCATTGC		

### DNA Microarray and Data Analysis

#### RNA Extraction, Target Labeling, Hybridization, Scanning, data processing and value definition

All data and protocols were assigned GEO accession numbers as appended below: (GSE61498, http://www.ncbi.nlm.nih.gov/geo/query/acc.cgi?acc=GSE61498) All steps from RNA amplification to the final scanner output were conducted by a private contractor (Biochip, Shanghai, China). The main gene symbols and gene descriptions were listed in Table 1.

#### Microarray Data Analysis

Bioinformatic analyses including molecular function, biological processes, cellular components, and KEGG pathway were conducted using MAS 3.0 molecule annotation system, DAVID Bioinformatics Resources 6.7, Amigo, GeneInfoViz Constructing and Visualizing Gene Relation Networks, and SBC analysis system [9, 35]

### Statistical Analysis

All results are presented as the mean ± SD. Statistical analysis was performed by one-way analysis of variance (ANOVA) and post hoc tests using SPSS version 17.0 (SPSS, Inc., an IBM Company, Chicago, IL, USA). A P-value of < 0.05 was considered significant.

## Results

### Assessment of the Stress Model

**Fig 1:**
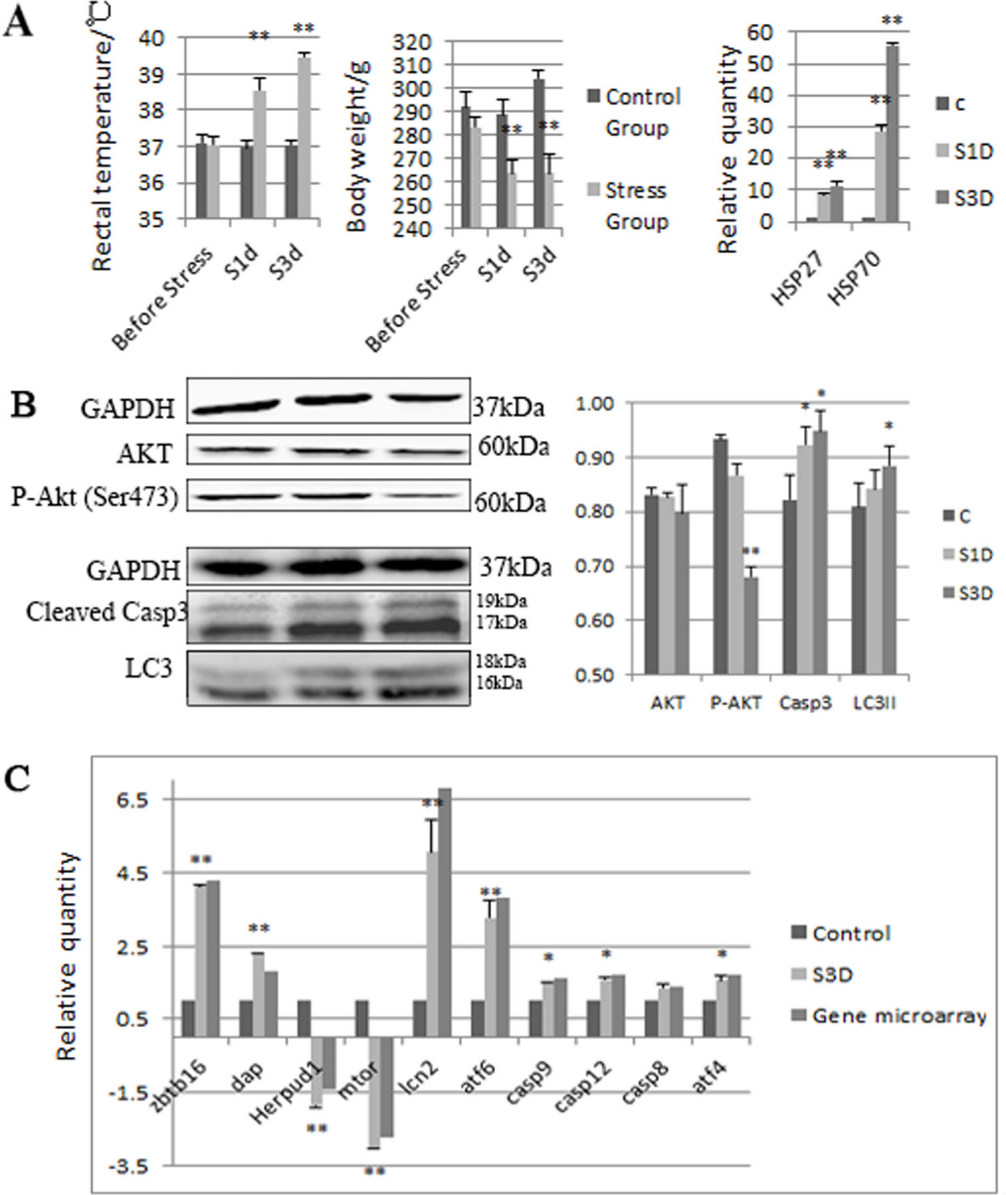
Heat- and shake-induced stress response in rats. (A) Heat- and shake-induced stress response in rats. (A) Stress induced temperature and weight changes (n = 6), and the level of HSP27 and 70 (n = 3) detected by RT-PCR in rats. Values represent the mean ± SD rats for each group. (B) Quantification of western blot determination of P-AKT, AKT, cleaved Casp3 and LC3; (C) mRNA expression levels of Casp12, Casp8, Casp9, Atf4, Atf6, Lcn2, Dap, Zbtb16, Herpud1, and mTOR in the rat jejunum were quantified by real-time PCR. Sections of small intestine were collected from control, 1-day, or 3-day stressed rats. P-AKT and AKT were significantly decreased after 1-day or 3-day stress. Casp12, Casp8, Casp9, Atf4, Atf6, Lcn2, Dap, Zbtb16, and Atf6 levels were significantly increased, while Herpud1 and mTOR levels were decreased. Values are expressed as a percentage of control. Data are mean ± SE, n = 3 rats for each group. *P < 0.05, **P < 0.01 compared with control; t-test.

The small intestine tissue (jejunum) obtained from rats on the first and third day of treatment was used for the molecular experiments. The rectal temperatures of rats were significantly increased after the stress. The body weights were decreased notably compared with the control group. Moreover, the mRNA expression levels of Hsp27 and Hsp70 were significantly increased compared with the control, (P < 0.01; Fig 1A)

### Pathological and Histological Changes in the Small Intestine after Stress in Rats

**Fig 2:**
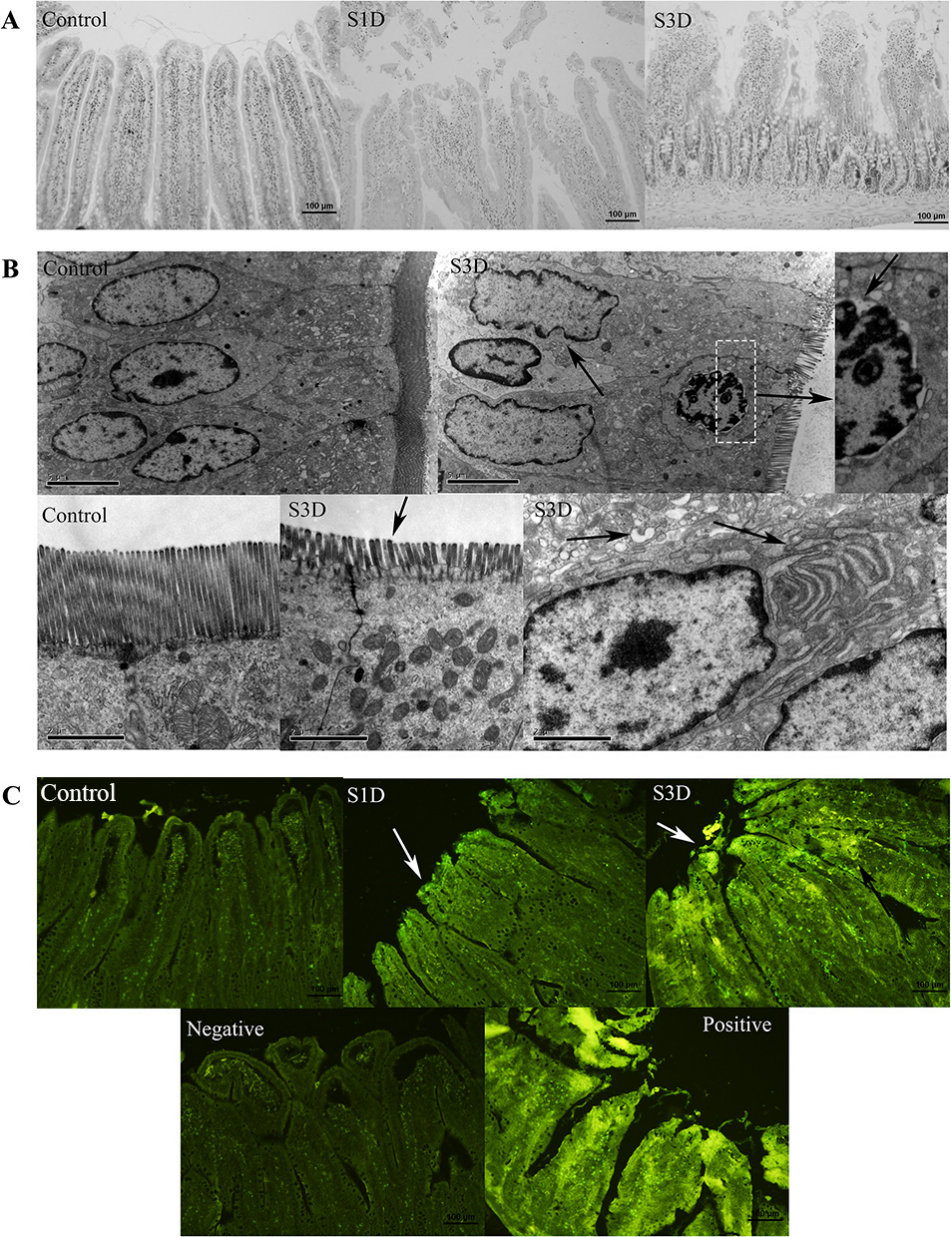
Histological changes in the small intestine following transport-associated stress. (A) Photomicrographs of H&E-stained sections of rat jejunum from the control, 1-day, and 3-day stress groups. (B) Morphological alterations in the ultrastructure of rat jejunal epithelium following treatment in the 3-day stress group. (C) Fluorescent microscopic images of TUNEL-stained sections of rat jejunum from the control, 1-day, and 3-day stress groups. The negative control was incubated without the TUNEL reaction mixture. The positive control was incubated with micrococcal nuclease to induce DNA breaks prior to the labeling procedures.

**Fig 3:**
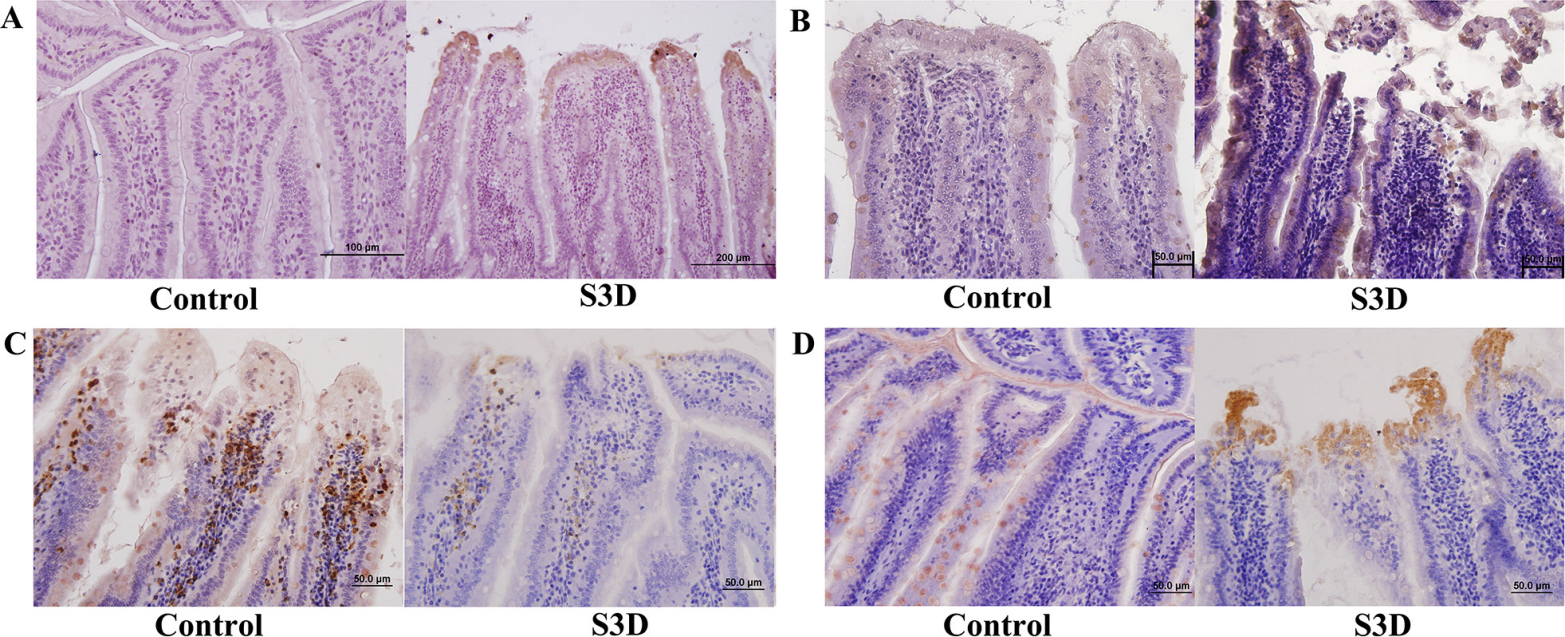
ER stress and autophagy activity in rat intestine. (A): LC3, (B): Casp3, (C): mTOR and (D): Casp12 expression in the small intestine (jejunal villus) of rats subjected to 3 days of stress treatment or control conditions. LC3, Casp3, and Casp12 levels increased while mTOR levels decreased (brown stain; sections counterstained with hematoxylin, light purple) in response to heat treatment above control levels.

Heat- and Shake- stress led to a series of clinical symptoms including fatigue and diarrhea. The rats’ hair was wet and unkempt because rats don’t sweat. They coated themselves in saliva in response to heat stress and attempt to cool themselves down. Necropsy showed that the serosa was hyperemic with intestinal vascular engorgement after simulated stress. To study the morphological changes, paraffin sections of jejunum were stained with hematoxylin and eosin. The epithelial cells at the villus tip were shedding and the lamina propria was exposed in the two stress groups, most severely in the S3d group, which suffered more stress than in the other groups (Fig 2A). To further study the ultrastructural changes, the jejunum of the S3d group was examined using transmission electron microscopy (Fig 2B). TUNEL staining showed increased positive substances, which indicated that apoptosis increased at the top of the jejunal villi and transferred to the basement membrane in the stress groups (Fig 2C). The results of the immunohistochemistry tests also showed the structure damage at the top of jejunal villi. Besides, Casp3, Casp12, LC3 and mTOR were changed at the villus tips (Fig 3). Photomicrographs revealed that the microvilli of the intestinal epithelium were atrophying and became shorter and sparser after the stress. The nuclei of the columnar epithelial cells in the stress groups were morphologically abnormal. The nuclear envelope invaginated in a serrated shape, and gaps in the karyolemma increased, revealing early symptoms of apoptosis and necrosis. The ER swelled significantly, indicating ER stress in the cells. These results indicated that the stress caused severe jejunal damage.

### mRNA Expression Profiling and Bioinformatics Analysis of Differentially Expressed Genes Related to Apoptosis and Autophagy

Gene expression profiling of the rats’ jejunum was performed by DNA microarrays using samples from the Sd3 group and controls. More than 41 000 rat genes and transcripts were investigated. Because apoptosis and autophagy induced by ER stress may be the potential mechanism of jejunal injury in the stress, 93 apoptosis or autophagy-related differentially expressed genes were selected (Table 3) and 67 genes such as Os9, Casp12, Atf4, Col4a3bp, Ddit3, Atg10, and Scamp5 in ER unfolded protein response (GO:0030968) were up-regulated, suggesting that ER stress was activated. At the same time, Atg4b, Wipi1, Arsa, Atg7, Atg10, Lamp1, Map1lc3b, Ctsd, Atg5, Tm9sf1, and Irgm in autophagy (GO:0006914) increased, also suggesting that autophagy was activated. Thirty-one genes including Bcl2ls, Eif2s, mTOR (P<0.01 and fold-change ≥ 1.5) were down-regulated, potentially indicating that a related signaling pathway may be involved in the stress response.

**Table 3:** Fold changes in the differentially expressed genes.

Gene Symbol	Fold change	Description	Genbank Accession	PValues
Naip5	10.28	Neuronal apoptosis inhibitory protein Fragment [Source:UniProtKB/TrEMBL;Acc:Q8R4U8] [ENSRNOT00000061195]	XM_001070842	2.93E-03
Lcn2	6.84	Rattus norvegicus lipocalin 2 (Lcn2), mRNA [NM_130741]	NM_130741	1.89E-03
Bcl2l1	6.64	Rattus norvegicus Bcl2-like 1 (Bcl2l1), nuclear gene encoding mitochondrial protein, transcript variant 2, mRNA [NM_031535]	NM_031535	1.19E-05
Creb3l2	4.69	Rattus norvegicus cAMP responsive element binding protein 3-like 2 (Creb3l2), mRNA [NM_001012188]	NM_001012188	1.32E-02
Naip2	4.37	Rattus norvegicus mRNA for neuronal inhibitor of apoptosis (NAIP gene). [AJ271303]	AJ271303	1.82E-03
Zbtb16	4.36	Rattus norvegicus zinc finger and BTB domain containing 16 (Zbtb16), mRNA [NM_001013181]	NM_001013181	3.68E-03
Atf6	3.79	Rattus norvegicus activating transcription factor 6 (Atf6), mRNA [NM_001107196]	NM_001107196	2.90E-05
Bcl2l11	3.02	Rattus norvegicus BCL2-like 11 (apoptosis facilitator) (Bcl2l11), transcript variant 3, mRNA [NM_171988]	NM_171988	5.30E-04
Mmp28	2.95	Rattus norvegicus matrix metallopeptidase 28 (Mmp28), mRNA [NM_001079888]	NM_001079888	5.86E-04
Hspa9	2.94	Rattus norvegicus heat shock protein 9 (Hspa9), nuclear gene encoding mitochondrial protein, mRNA [NM_001100658]	NM_001100658	1.27E-03
Os9	2.73	Rattus norvegicus osteosarcoma amplified 9 (Os9), mRNA [NM_001007265]	NM_001007265	6.94E-05
Ddit4l	2.64	Rattus norvegicus DNA-damage-inducible transcript 4-like (Ddit4l), mRNA [NM_080399]	NM_080399	1.34E-02
Siva1	2.60	Rattus norvegicus SIVA1, apoptosis-inducing factor (Siva1), mRNA [NM_001100982]	NM_001100982	4.70E-04
Syvn1	2.54	Rattus norvegicus synovial apoptosis inhibitor 1, synoviolin (Syvn1), mRNA [NM_001100739]	NM_001100739	2.44E-04
RGD1306565	2.48	PREDICTED: Rattus norvegicus similar to apoptosis signal-regulating kinase 1 (RGD1306565), partial mRNA [XM_344798]	XM_344798	4.32E-03
Hspa1b	2.34	Rattus norvegicus heat shock 70kD protein 1B (mapped) (Hspa1b), mRNA [NM_212504]	NM_212504	1.75E-01
Gadd45g	2.31	Rattus norvegicus growth arrest and DNA-damage-inducible, gamma (Gadd45g), mRNA [NM_001077640]	NM_001077640	3.51E-03
Mmp7	2.19	Rattus norvegicus matrix metallopeptidase 7 (Mmp7), mRNA [NM_012864]	NM_012864	2.31E-02
Nyw1	2.19	Rattus norvegicus ischemia related factor NYW-1 (Nyw1), mRNA [NM_001134341]	NM_001134341	2.58E-02
Cflar	2.13	Rattus norvegicus CASP8 and FADD-like apoptosis regulator (Cflar), transcript variant 2, mRNA [NM_057138]	NM_057138	5.50E-03
Hspa1a	2.12	Rattus norvegicus heat shock 70kD protein 1A (Hspa1a), mRNA [NM_031971]	NM_031971	1.32E-01
Bik	2.07	Rattus norvegicus BCL2-interacting killer (apoptosis-inducing) (Bik), mRNA [NM_053704]	NM_053704	1.28E-03
Atg4b	2.01	Rattus norvegicus ATG4 autophagy related 4 homolog B (S. cerevisiae) (Atg4b), mRNA [NM_001025711]	NM_001025711	1.33E-03
Mmp19	1.99	Rattus norvegicus matrix metallopeptidase 19 (Mmp19), mRNA [NM_001107159]	NM_001107159	1.08E-01
Capn1	1.91	Rattus norvegicus calpain 1 (Capn1), mRNA [NM_019152]	NM_019152	6.70E-06
Psen1	1.88	Presenilin-1 (PS-1)(EC 3.4.23.-)(Protein S182) [Contains Presenilin-1 NTF subunit;Presenilin-1 CTF subunit;Presenilin-1 CTF12(PS1-CTF12)] [Source:UniProtKB/Swiss-Prot;Acc:P97887] [ENSRNOT00000012495]	FQ231303	4.61E-04
Aen	1.86	Rattus norvegicus apoptosis enhancing nuclease (Aen), mRNA [NM_001108487]	NM_001108487	2.95E-04
Dap	1.81	Rattus norvegicus death-associated protein (Dap), mRNA [NM_022526]	NM_022526	4.20E-02
Traf3	1.78	Rattus norvegicus Tnf receptor-associated factor 3 (Traf3), mRNA [NM_001108724]	NM_001108724	7.90E-05
Casp12	1.77	Rattus norvegicus caspase 12 (Casp12), mRNA [NM_130422]	NM_130422	8.58E-03
Wipi1	1.75	Rattus norvegicus WD repeat domain, phosphoinositide interacting 1 (Wipi1), mRNA [NM_001127297]	NM_001127297	2.55E-02
Bak1	1.74	Rattus norvegicus BCL2-antagonist/killer 1 (Bak1), mRNA [NM_053812]	NM_053812	3.00E-05
Arsa	1.70	Rattus norvegicus arylsulfatase A (Arsa), mRNA [NM_001034933]	NM_001034933	3.21E-04
Chac1	1.68	Rattus norvegicus ChaC, cation transport regulator homolog 1 (E. coli) (Chac1), mRNA [NM_001173437]	NM_001173437	1.34E-01
Gadd45b	1.68	Rattus norvegicus growth arrest and DNA-damage-inducible, beta (Gadd45b), mRNA [NM_001008321]	NM_001008321	5.32E-03
Atf4	1.67	Rattus norvegicus activating transcription factor 4 (tax-responsive enhancer element B67) (Atf4), mRNA [NM_024403]	NM_024403	5.78E-04
Atg7	1.67	Rattus norvegicus ATG7 autophagy related 7 homolog (S. cerevisiae) (Atg7), mRNA [NM_001012097]	NM_001012097	2.43E-04
Col4a3bp	1.66	Rattus norvegicus collagen, type IV, alpha 3 (Goodpasture antigen) binding protein (Col4a3bp), mRNA [NM_001108935]	NM_001108935	4.56E-03
Aifm2	1.65	Rattus norvegicus apoptosis-inducing factor, mitochondrion-associated 2 (Aifm2), nuclear gene encoding mitochondrial protein, mRNA [NM_001139483]	NM_001139483	7.20E-04
Casp9	1.59	Rattus norvegicus caspase 9, apoptosis-related cysteine peptidase (Casp9), mRNA [NM_031632]	NM_031632	7.05E-04
Casp2	1.59	Rattus norvegicus caspase 2 (Casp2), mRNA [NM_022522]	NM_022522	1.19E-03
Ddit3	1.59	Rattus norvegicus DNA-damage inducible transcript 3 (Ddit3), transcript variant 2, mRNA [NM_024134]	NM_024134	3.23E-04
Ciapin1	1.53	Rattus norvegicus cytokine induced apoptosis inhibitor 1 (Ciapin1), mRNA [NM_001007689]	NM_001007689	4.81E-03
Dap3	1.50	Rattus norvegicus death associated protein 3 (Dap3), nuclear gene encoding mitochondrial protein, mRNA [NM_001011950]	NM_001011950	1.59E-03
RGD1311605	1.49	Rattus norvegicus similar to apoptosis related protein APR-3; p18 protein (RGD1311605), mRNA [NM_001127526]	NM_001127526	5.54E-05
Bag4	1.48	Rattus norvegicus BCL2-associated athanogene 4 (Bag4), mRNA [NM_001025130]	NM_001025130	2.22E-04
Thap1	1.48	Rattus norvegicus THAP domain containing, apoptosis associated protein 1 (Thap1), mRNA [NM_001008340]	NM_001008340	1.29E-02
Atg10	1.47	Rattus norvegicus autophagy-related 10 (S. cerevisiae) (Atg10), mRNA [NM_001109505]	NM_001109505	1.12E-03
Traf7	1.46	Rattus norvegicus Tnf receptor-associated factor 7 (Traf7), mRNA [NM_001127548]	NM_001127548	1.17E-03
Lamp1	1.45	Rattus norvegicus lysosomal-associated membrane protein 1 (Lamp1), mRNA [NM_012857]	NM_012857	1.07E-04
Xiap	1.41	Rattus norvegicus X-linked inhibitor of apoptosis (Xiap), mRNA [NM_022231]	NM_022231	4.20E-03
Tsc2	1.41	Rattus norvegicus tuberous sclerosis 2 (Tsc2), mRNA [NM_012680]	NM_012680	1.16E-04
Casp8	1.40	Rattus norvegicus caspase 8 (Casp8), mRNA [NM_022277]	NM_022277	2.86E-03
Map1lc3b	1.35	Rattus norvegicus microtubule-associated protein 1 light chain 3 beta (Map1lc3b), mRNA [NM_022867]	NM_022867	2.41E-05
Ctsd	1.33	Rattus norvegicus cathepsin D (Ctsd), mRNA [NM_134334]	NM_134334	1.21E-02
Tsc1	1.27	Rattus norvegicus tuberous sclerosis 1 (Tsc1), mRNA [NM_021854]	NM_021854	1.48E-01
Hspb1	1.27	Rattus norvegicus heat shock protein 1 (Hspb1), mRNA [NM_031970]	NM_031970	1.03E-01
Atg5	1.20	Rattus norvegicus ATG5 autophagy related 5 homolog (S. cerevisiae) (Atg5), mRNA [NM_001014250]	NM_001014250	6.54E-02
Perp	1.20	Rattus norvegicus PERP, TP53 apoptosis effector (Perp), mRNA [NM_001106265]	NM_001106265	7.11E-03
Bad	1.16	Rattus norvegicus BCL2-associated agonist of cell death (Bad), mRNA [NM_022698]	NM_022698	5.37E-02
Bcl2	1.15	Rattus norvegicus B-cell CLL/lymphoma 2 (Bcl2), nuclear gene encoding mitochondrial protein, mRNA [NM_016993]	NM_016993	1.36E-01
Fam129a	1.11	Rattus norvegicus family with sequence similarity 129, member A (Fam129a), mRNA [NM_022242]	NM_022242	4.70E-01
Scamp5	1.07	Rattus norvegicus secretory carrier membrane protein 5 (Scamp5), mRNA [NM_031726]	NM_031726	5.13E-02
Atg12	1.06	Rattus norvegicus ATG12 autophagy related 12 homolog (S. cerevisiae) (Atg12), mRNA [NM_001038495]	NM_001038495	3.33E-01
Tm9sf1	1.05	Rattus norvegicus transmembrane 9 superfamily member 1 (Tm9sf1), mRNA [NM_001012155]	NM_001012155	1.41E-01
Irgm	1.02	Rattus norvegicus immunity-related GTPase family, M (Irgm), mRNA [NM_001012007]	NM_001012007	7.92E-01
Becn1	1.02	Rattus norvegicus beclin 1, autophagy related (Becn1), transcript variant 1, mRNA [NM_053739]	NM_053739	5.07E-01
Map1lc3a	0.93	Rattus norvegicus microtubule-associated protein 1 light chain 3 alpha (Map1lc3a), mRNA [NM_199500]	NM_199500	2.83E-01
Eif2ak2	0.89	Rattus norvegicus eukaryotic translation initiation factor 2-alpha kinase 2 (Eif2ak2), mRNA [NM_019335]	NM_019335	1.22E-01
Mmp9	0.86	Rattus norvegicus matrix metallopeptidase 9 (Mmp9), mRNA [NM_031055]	NM_031055	3.78E-01
RGD1359310	0.84	Rattus norvegicus similar to RIKEN cDNA 9430023L20 (RGD1359310), mRNA [NM_001007659]	NM_001007659	3.28E-02
Eif2b1	0.80	Rattus norvegicus eukaryotic translation initiation factor 2B, subunit 1 alpha (Eif2b1), mRNA [NM_172029]	NM_172029	4.65E-02
Triap1	0.80	Rattus norvegicus TP53 regulated inhibitor of apoptosis 1 (Triap1), mRNA [NM_001126099]	NM_001126099	2.47E-03
Aifm1	0.80	Rattus norvegicus apoptosis-inducing factor, mitochondrion-associated 1 (Aifm1), nuclear gene encoding mitochondrial protein, mRNA [NM_031356]	NM_031356	4.26E-04
Eif2ak1	0.79	Rattus norvegicus eukaryotic translation initiation factor 2 alpha kinase 1 (Eif2ak1), mRNA [NM_013223]	NM_013223	1.21E-02
Arsb	0.79	Rattus norvegicus arylsulfatase B (Arsb), mRNA [NM_033443]	NM_033443	6.28E-02
Eif4ebp2	0.75	Rattus norvegicus eukaryotic translation initiation factor 4E binding protein 2 (Eif4ebp2), mRNA [NM_001033069]	NM_001033069	2.34E-02
Herpud1	0.75	Rattus norvegicus homocysteine-inducible, endoplasmic reticulum stress-inducible, ubiquitin-like domain member 1 (Herpud1), mRNA [NM_053523]	NM_053523	2.46E-02
Ccar1	0.68	Rattus norvegicus cell division cycle and apoptosis regulator 1 (Ccar1), mRNA [NM_001108535]	NM_001108535	1.31E-03
Nol3	0.63	Rattus norvegicus nucleolar protein 3 (apoptosis repressor with CARD domain) (Nol3), mRNA [NM_053516]	NM_053516	3.99E-03
Fas	0.59	Rattus norvegicus Fas (TNF receptor superfamily, member 6) (Fas), mRNA [NM_139194]	NM_139194	1.57E-04
Api5	0.57	Rattus norvegicus apoptosis inhibitor 5 (Api5), mRNA [NM_001127379]	NM_001127379	7.82E-04
Bcl2l2	0.57	Rattus norvegicus Bcl2-like 2 (Bcl2l2), mRNA [NM_021850]	NM_021850	4.53E-04
Aven	0.56	Rattus norvegicus apoptosis, caspase activation inhibitor (Aven), mRNA [NM_001107757]	NM_001107757	2.83E-04
Eif2ak4	0.54	eukaryotic translation initiation factor 2 alpha kinase 4 [Source:RefSeq peptide;Acc:NP_001099214] [ENSRNOT00000009222]	FQ228781	8.38E-05
Atp2a1	0.52	Rattus norvegicus ATPase, Ca++ transporting, cardiac muscle, fast twitch 1 (Atp2a1), mRNA [NM_058213]	NM_058213	3.81E-03
Eif2a	0.52	Rattus norvegicus eukaryotic translation initiation factor 2A (Eif2a), mRNA [NM_001109339]	NM_001109339	3.06E-04
Bid	0.51	Rattus norvegicus BH3 interacting domain death agonist (Bid), mRNA [NM_022684]	NM_022684	2.04E-03
Bcl2l10	0.49	Rattus norvegicus BCL2-like 10 (apoptosis facilitator) (Bcl2l10), mRNA [NM_053733]	NM_053733	3.13E-03
Mtor	0.44	Rattus norvegicus mechanistic target of rapamycin (serine/threonine kinase) (Mtor), mRNA [NM_019906]	NM_019906	6.92E-04
Bcl2l14	0.43	Rattus norvegicus Bcl2-like 14 (apoptosis facilitator) (Bcl2l14), mRNA [NM_001024338]	NM_001024338	2.24E-03
Eif2ak3	0.37	Rattus norvegicus eukaryotic translation initiation factor 2 alpha kinase 3 (Eif2ak3), mRNA [NM_031599]	NM_031599	9.00E-04
Bfar	0.27	Rattus norvegicus bifunctional apoptosis regulator (Bfar), mRNA [NM_001013125]	NM_001013125	8.95E-05

**Fig 4:**
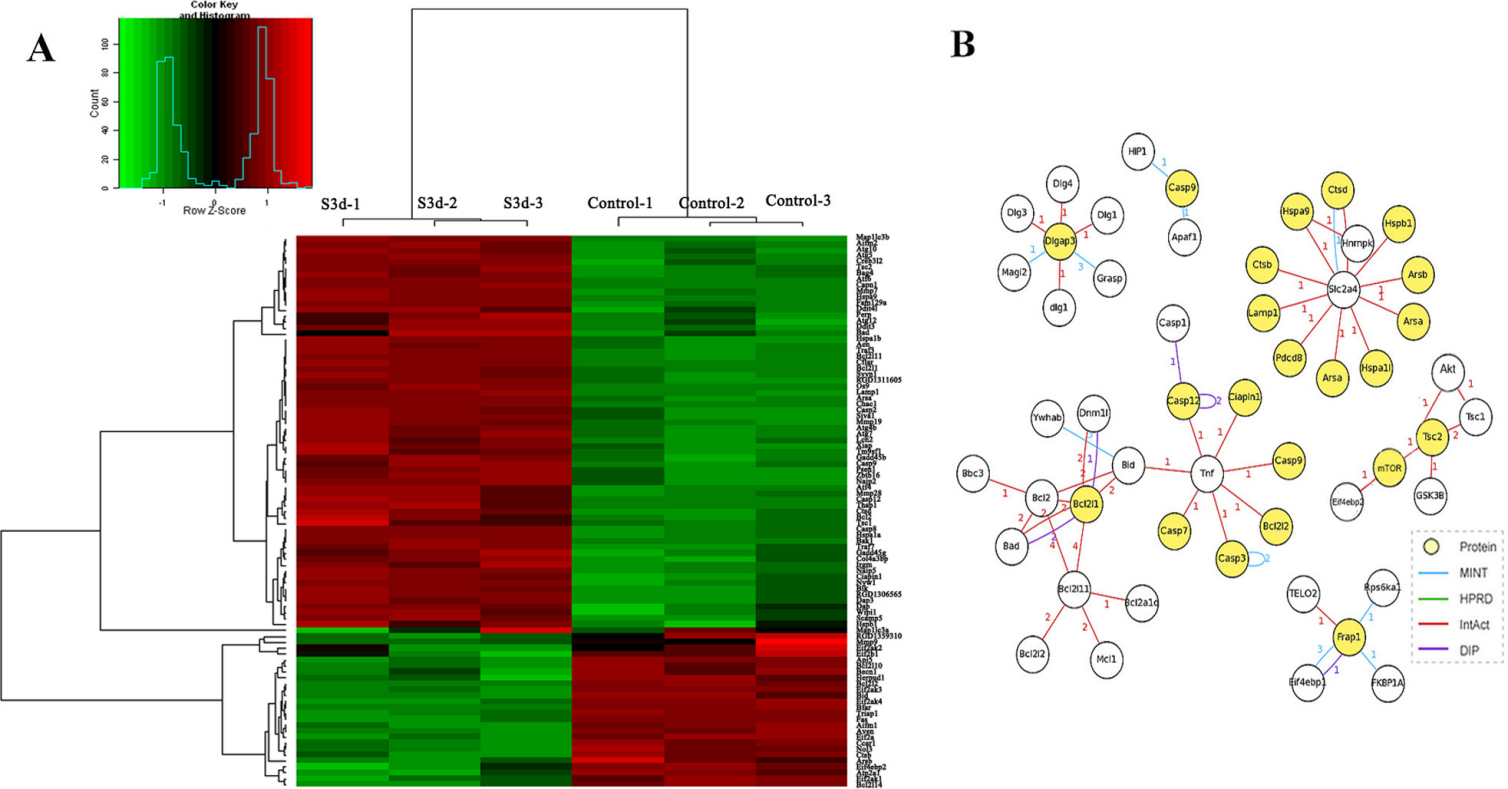
(A) The heat map shows differentially expressed genes in the control and 3-day stress groups. Three samples were included in each group and the gene expression profiles are shown in rows. Red and green in the heat map represent up-regulation and down-regulation relative to the control, respectively. (B) Gene interaction network map of ER stress-related molecules including mTOR, Tnf, Casp12, Bcl2ls, Slc2a4 and others using MAS 3.0 based on the database.

**Fig 5:**
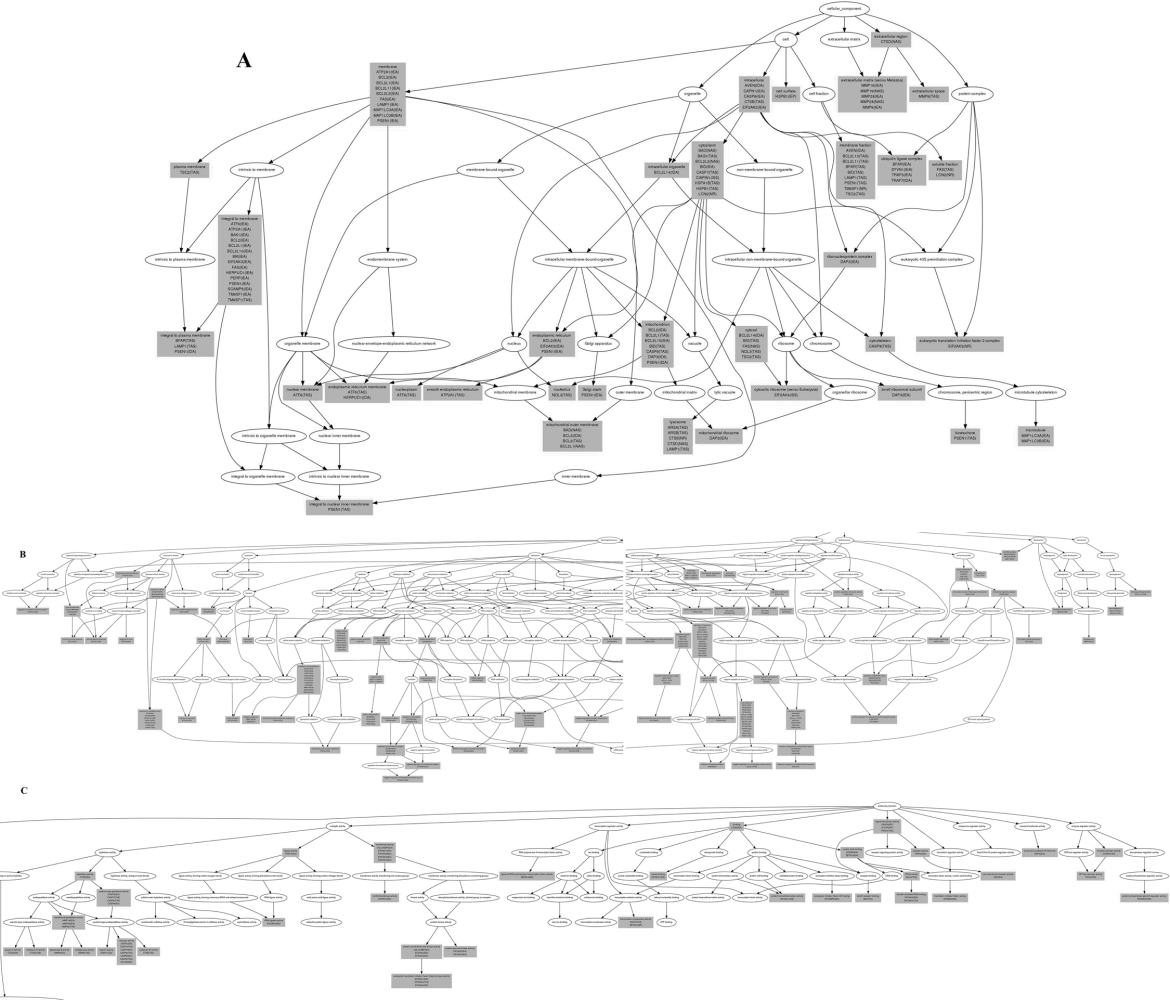
GO term enrichment graph, construction and dynamic visualization of the gene relation network. (A) Cellular Components; 68 differentially expressed genes were related to 39 chart records in cellular components, mainly enriched in the GO related to the cell membranes, such as the organelle membrane, organelle envelope, membrane-enclosed lumen, ER, cytoplasmic vesicle, and autophagic vacuole (B) Biological Processes; 15 genes were related to autophagy, 24 to the cellular response to stress, and 11 to the response to endoplasmic reticulum stress (C) Molecular Function. There were also 22 chart records in molecular function, mainly enriched in the GO related to protein binding and calcium ion binding.

**Fig 6:**
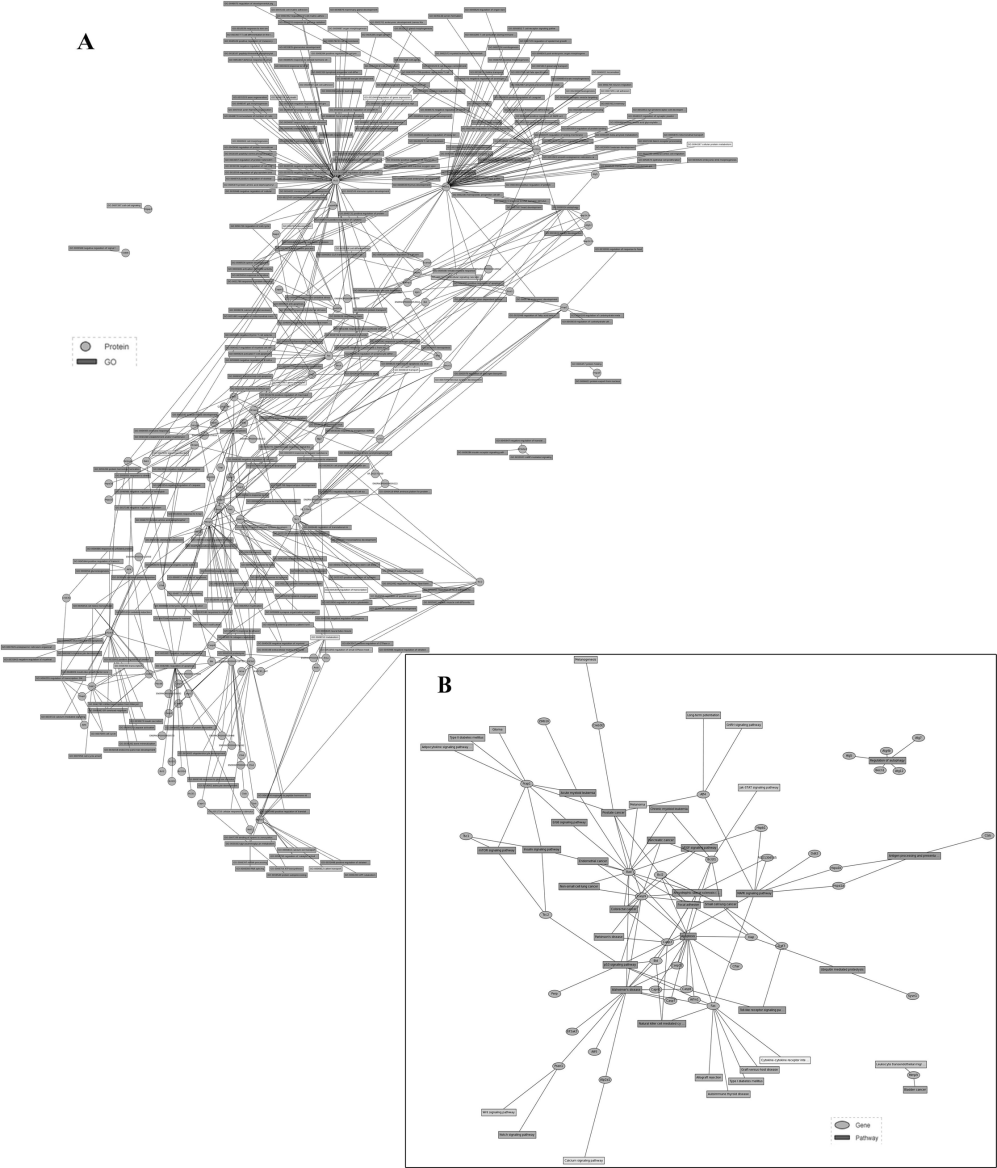
The network graph of proteins and GO, and the gene pathway network graph (A) GO protein network graph of the biological processes using MAS 3.0. (B) Gene pathway network graph of the biological processes based on KEGG using MAS 3.0.

**Fig 7:**
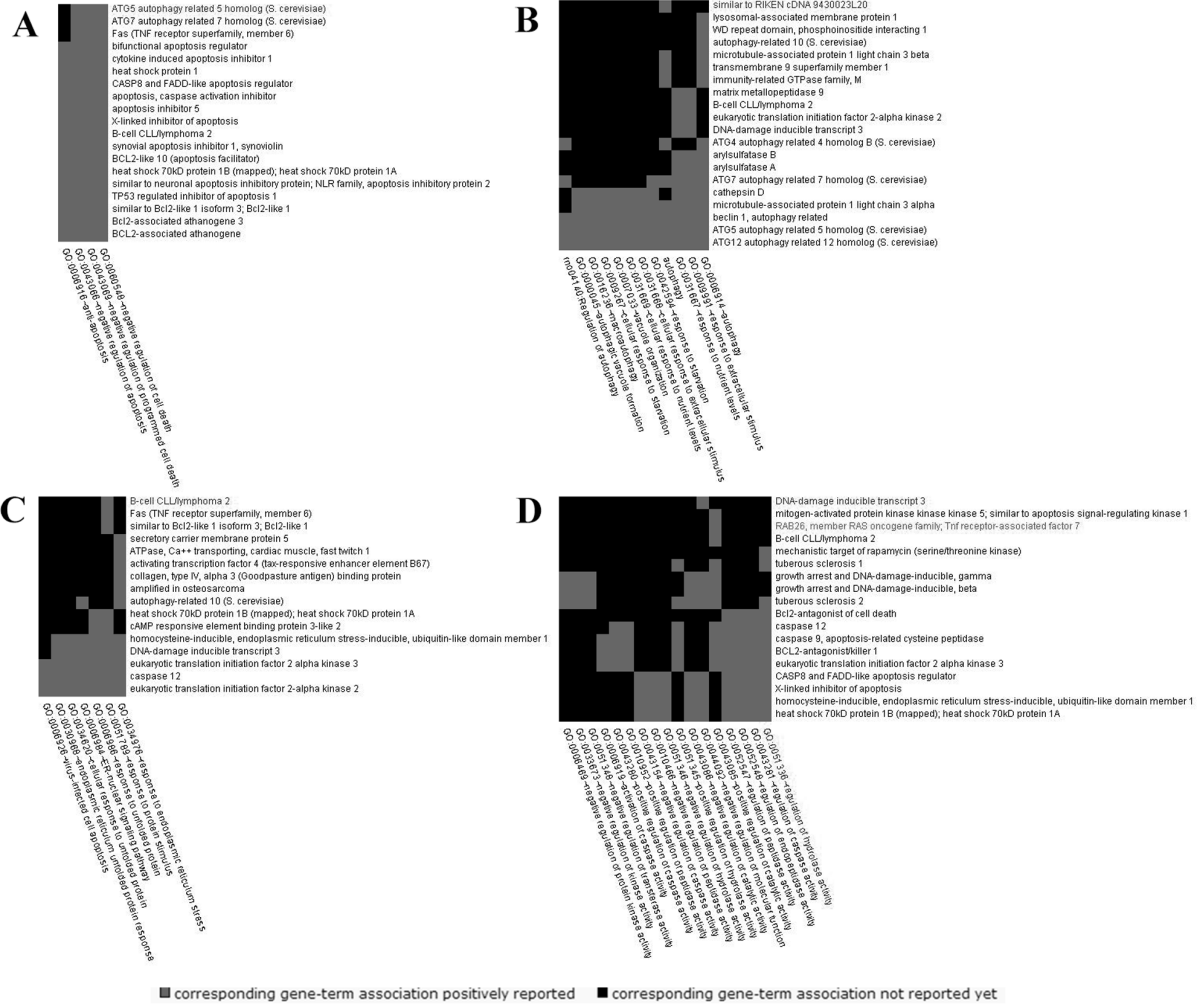
2D view of the functional annotation clustering using DAVID software (A) Cluster of anti-apoptosis-related GO (B) Cluster of autophagy-related GO; (C) Cluster of ER stress-related GO (D) Cluster of autophagic vacuole-related GO.

The 93 differentially expressed genes were analyzed by a Molecule Annotation System, MAS 3.0, GeneInfoViz and DAVID, and were classified into GO slim terms. Table 3 shows these differentially expressed genes in more detail. Heat map was conducted showed visualize differentially expressed genes between the control group and S3d the (Fig 4A) The distribution in three major gene ontologies: biological processes, cellular components, and molecular function were showed. Eighty-five differentially expressed genes were related to 267 chart records in biological processes, 41 genes were enriched in the regulation of apoptosis (GO:0042981, GO:0043067, GO:0010941), and half were related to the positive regulation of apoptosis (GO:0043065, GO:0043068, GO:0010942). Another 19 genes each for negative regulation (GO:0043066, GO:0043069, GO:0060548). Also, 15 genes were related to autophagy (GO:0006914), 23 to the cellular response to stress (GO:0033554), and 11 to the response to endoplasmic reticulum stress (GO:0034976) (Fig 5B). Sixty-eight differentially expressed genes were related to 39 chart records in cellular components, mainly enriched in the GO related to the cell membranes, such as the organelle membrane, organelle envelope, membrane-enclosed lumen, ER, cytoplasmic vesicle, autophagic vacuole and others (GO:0031090, GO:0031967, GO:0031974, GO:0005783, GO:0031410, and GO:0005776, respectively) (Fig 5A). There were also 22 chart records in molecular function, mainly enriched in the GO related to protein binding and calcium ion binding (Fig 5C). Some of the altered GO are listed in Table 4. In GO cluster analysis, the black squares represented gene-term associations not yet reported, suggesting a potential new gene-term for differentially expressed genes in the stress. In the analysis of the comparison of the network graph of proteins and GO (Fig 6A) and the gene pathway network graph (Fig 6B), AKT1, TSC1/2, Casp9, Fas, Bcl2s, Bcl2ls, Eif-2s, Mmp9, Zbtb16, and others were important. The 2-D view module functional annotation clustering graphic presentations helped to understand the common biology among related genes in the anti-apoptosis related GO; enrichment score is 12.29. (Fig 7A), autophagy related GO; enrichment score is 5.44. (Fig 7B), ER stress related GO; enrichment score is 6.75 (Fig 7C) and autophagic vacuole related GO; enrichment score is 4.04 (Fig 7D) related GO. Also, the analysis pinpointed the key biological differences among related genes, providing links to detailed resources for drill-down analysis. To define the biological pathways related to Heat- and Shake- stress in rat jejunum, we used MAS 3.0 (http://bioinfo.capitalbio.com/mas3/) and a Molecule Annotation System (http://sas.ebioservice.com/uiframe.firstpage.do) and KEGG pathway analysis revealed that 14 genes were in the apoptosis pathway, nine were in the p53 signaling pathway, five were involved in the regulation of autophagy, nine in the MAPK signaling pathway, and three in the mTOR signaling pathway (Table 5).

**Table 4:** GO analysis of the differentially expressed genes.

Category	Term	Count	%	PValue	Fold Enrichment
Biological processes	GO:0042981~regulation of apoptosis	41	48.31	1.33E-30	9.04
	GO:0043067~regulation of programmed cell death	41	48.31	2.27E-30	8.92
	GO:0010941~regulation of cell death	41	48.31	2.72E-30	8.88
	GO:0033554~cellular response to stress	23	26.97	1.21E-14	7.83
	GO:0043065~positive regulation of apoptosis	19	23.60	6.52E-14	9.05
	GO:0043068~positive regulation of programmed cell death	19	23.60	7.31E-14	9.00
	GO:0010942~positive regulation of cell death	19	23.60	8.66E-14	8.92
	GO:0043066~negative regulation of apoptosis	19	22.47	8.92E-13	8.54
	GO:0043069~negative regulation of programmed cell death	19	22.47	1.16E-12	8.42
	GO:0060548~negative regulation of cell death	19	22.47	1.22E-12	8.39
	GO:0009719~response to endogenous stimulus	17	19.10	1.67E-06	4.22
	GO:0006916~anti-apoptosis	16	17.98	5.34E-14	15.70
	GO:0006914~autophagy	15	16.85	1.76E-24	82.07
	GO:0006917~induction of apoptosis	12	13.48	8.22E-08	8.89
	GO:0012502~induction of programmed cell death	12	13.48	8.22E-08	8.89
	GO:0034976~response to endoplasmic reticulum stress	11	12.36	1.72E-14	46.02
	GO:0051789~response to protein stimulus	10	11.24	1.76E-08	14.97
	GO:0042127~regulation of cell proliferation	10	11.24	0.040708	2.14
	GO:0001666~response to hypoxia	6	7.87	0.002103	5.21
	GO:0006979~response to oxidative stress	5	6.74	0.008857	4.66
	GO:0007584~response to nutrient	6	6.74	0.014243	4.14
Cellular components	GO:0031090~organelle membrane	19	21.35	1.13E-05	3.20
	GO:0031967~organelle envelope	13	14.61	1.20E-04	3.77
	GO:0031975~envelope	13	14.61	1.29E-04	3.74
	GO:0031974~membrane-enclosed lumen	11	14.61	0.08433	1.64
	GO:0005773~vacuole	11	12.36	3.33E-07	8.85
	GO:0005783~endoplasmic reticulum	11	12.36	0.020355	2.26
	GO:0031410~cytoplasmic vesicle	10	11.24	0.010937	2.66
	GO:0031968~organelle outer membrane	9	10.11	1.36E-07	14.70
	GO:0019867~outer membrane	9	10.11	1.86E-07	14.12
	GO:0005792~microsome	7	7.87	0.005635	4.23
	GO:0005764~lysosome	6	6.74	0.003291	5.87
	GO:0005776~autophagic vacuole	5	5.62	9.36E-07	60.95
	GO:0044432~endoplasmic reticulum part	5	5.62	0.079853	3.02
	GO:0000421~autophagic vacuole membrane	4	4.49	4.53E-06	105.65
Molecular function	GO:0042802~identical protein binding	14	15.73	2.30E-06	5.00
	GO:0070011~peptidase activity, acting on L-amino acid peptides	13	14.61	4.29E-06	5.20
	GO:0004175~endopeptidase activity	12	13.48	1.10E-06	6.68
	GO:0005509~calcium ion binding	7	7.87	0.092853	2.19

**Table 5:** KEGG pathway analysis of the differentially expressed genes.

Category	Term	Count	%	PValue	Fold Enrichment
KEGG_PATHWAY	rno04210:Apoptosis	12	15.73	3.20E-14	20.46
	rno05200:Pathways in cancer	13	14.61	3.66E-06	5.09
	rno05010:Alzheimer's disease	12	13.48	2.18E-07	7.57
	rno04115:p53 signaling pathway	9	10.11	3.10E-08	16.94
	rno04010:MAPK signaling pathway	9	10.11	9.41E-04	4.20
	rno05014:Amyotrophic lateral sclerosis (ALS)	8	8.99	2.86E-07	16.84
	rno05215:Prostate cancer	6	6.74	6.40E-04	8.28
	rno04140:Regulation of autophagy	5	5.62	5.96E-05	22.18
	rno04142:Lysosome	5	5.62	0.012989	5.31
	rno04150:mTOR signaling pathway	3	3.37	0.064802	7.03

We predicted that the interaction of molecules related to ER stress, apoptosis, and autophagy detected in the current study would provide insight into the possible mechanisms of heat and shaking stress-induced cellular damage. The key molecules may include mTOR, Tnf, Casp12, Bcl2ls, Slc2a4 and others (Fig 4B). The mTOR signaling pathway may be a response to heat and shake stress, because mTOR and eukaryotic translation initiation factor-2s (Eif2s) decreased while TSC1 and TSC2 increased, which may be negatively regulated by ER stress, a potential mechanism of enhanced autophagy.

Casp12, Casp8, Casp9, Atf4, Lcn2, Dap, Zbtb16, Atf6, Herpud1, and mTOR were detected by real-time PCR to verify differentially expressed genes in the gene microarray analysis. The mRNA expression levels of mTOR and Herpud1 were significantly down-regulated and the other genes were up-regulated in the rat jejunum after stress (P < 0.05; Fig 1C). The changes in gene expression closely correlated with the corresponding microarray data, although the exact fold-change differed between the two assays.

### Heat- and Shake- Stress Triggered ER Stress, Apoptosis, and Autophagy at the Top of the Rat Jejunal Villi

ER stress may induce apoptosis or autophagy during the stress process. The results of the immunohistochemistry tests showed that at the top of jejunal villi, Casp3, Casp12, and LC3 significantly increased while mTOR decreased (Figs 1 and 3). The western blot tests used whole jejunal tissue and revealed the level of proteins including AKT and P-AKT decreased compared with the control group (P < 0.05; Fig 1B). These results indicated that rat jejunum in the stress suffered ER stress and autophagy, especially at the top of the villi, and that apoptosis may concentrate at the top of the villi, rather than throughout the jejunal tissue.

## Discussion

### Heat- and Shake- Stress Induced Significant Damage to the Rat Jejunum

Heat stress occurs frequently in the summer, and shaking increases body temperature during transport. Both have effects in animals and humans, potentially leading to irritable bowel syndrome. The model in this study was applied to rats that were treated as follows: shaking at 0.1 × g to simulate shaking stress, a 35°C ambient temperature on a constant temperature shaker, for 2 h/d for 1 or 3 days, according to the group. The observed significant decreases in body weight and increases in rectal temperature, and transcription of hsp27/70confirmed successful stress responses in the rats. Small intestine tissue (jejunum) obtained from rats on the first and third day of treatment was used for molecular biology experiments and gene microarray.

The effects of stress resulting from transport and handling during shipping have been shown to impair the intestinal barrier [36]. In heat stress, compensatory mechanisms are activated to protect vital organs and dissipate internal heat at the body surface, reducing blood flow to the GI tract [2], leading to significant mucosal injury in the small intestine [37]. The abnormal clinical behavior we saw in this study reflected the rats’ physiological stress. The state of the intestine in the S3d group at necropsy showed a redistribution of blood flow. The intestinal barrier protects the body from pathogens and cytotoxic chemicals and because the epithelial cells at the villus tip were shedding and the lamina propria was exposed in the stressed rats, the competence of the intestinal barrier disintegrated (Fig 2A), leading to intestinal barrier dysfunction. We investigated the potential underlying mechanism, and found that the microstructural changes in the epithelial cells revealed apoptosis, seen as nuclear envelopes invaginating in a serrated pattern, increased gaps in the karyolemma, significant ER swelling, and nuclear pyknosis. These results were verified by TUNEL staining (Fig 2C). Apoptosis at the tip of villus increased and extended to the basement membrane of the villus. These results indicated that heat and shake stress induced significant damage to the rat jejunum, and may lead to diarrhea, inflammation, infection by microorganisms, or other GI disorders.

### Heat and Shake Stress Triggers ER Stress and Induced Apoptosis and Autophagy of the Rat Jejunum

Because of blood redistribution, ischemia of the intestine leads to reduced blood glucose in the jejunum [9]. Energy may then become limited for protein folding in the ER and therefore activate UPR [38]. Accumulation of misfolded proteins under stress also causes ER stress [39]. Swelling of the ER was seen in transmission electron microscopy in our study, indicating ER stress in rat jejunal epithelial cells after stress (Fig 2B).

In this study, whole jejunum tissues were used as the material for the gene microarray. In order to elucidate the stress response mechanism in vivo, Figs 5 and 6 were drawn to show the relationship between differentially expressed genes and the relevant GO and pathways. These results showed that ER stress, autophagy, apoptosis and other cellular functions are trigged in rat small intestine. The membrane is the main component of the ER and autophagy. According to GO term enrichment graph (Fig 5A) and Table 4, we found that differentially expressed genes were enriched in both the ER(GO:0005783) and autophagic vacuoles (GO:0005776) by the stress. They were also enriched in protein binding and calcium ion binding, which is the main function of the ER. ER stress triggers autophagy in mild stress [19]. However, if the stress is prolonged, or the adaptive response fails, ER stress triggers and initiates apoptosis [21]. Overall, our results indicated that ER stress plays a key role in heat and shake stress.

KEGG pathway analysis revealed that apoptosis, the p53 signaling pathway, autophagy, the MAPK signaling pathway, and the mTOR signaling pathway were involved in the mechanism of rat intestinal damage induced by heat and shake stress.

Our previous studies have shown that growth-related molecule expression was involved in heat stress-induced damage in rat jejunum based on gene microarray [5]. Oxidative stress and MAPK pathways were involved as well [8, 40]. Recently, several studies provided new insights into the function of the MAPK pathway in the control of the balance of autophagy and apoptosis in response to genotoxic stress [23]. Research has also shown that autophagy was involved in oxidative stress or simultaneously existed in certain pathological processes [41]. Oxidative stress and increased generation of ROS have been reported to serve as important stimuli of autophagy during periods of nutrient deprivation, ischemia/reperfusion, and in response to cell stress [42, 43]. Oxidative stress and MAPK signaling pathways were shown to be involved in heat stress-induced injury in the rat jejunum.

Autophagy is a method of cell survival and it plays an important role during the stress response. In nutrient limitation, ER stress, starvation, or other pathological conditions, autophagy breaks down unnecessary macromolecules and provides the intermediate metabolites of catabolism and anabolism [44–46]. During autophagy, the body must synthesize proteins with limited resources inside the cell; autophagy aids this by recycling nutrients. Therefore, autophagy is a basic means of cell survival in stress. In this study, LC3 significantly increased at the villus tip in the jejunum (Figs 1B and 3A), and in gene microarray analysis, approximately 10 differentially expressed genes involved in autophagy, including Atg4b, Arsa, Atg7, Atg10, Lamp1, Map1lc3b, Ctsd, Atg5, and others, were up-regulated. These results showed that autophagy increased in heat and shake stress. ER stress negatively regulates the AKT/TSC/mTOR pathway to enhance autophagy [30]. AKT, a serine/threonine-specific protein kinase, plays a fundamental role in cell survival and apoptosis [47]. mTOR inhibition induces upstream receptor tyrosine kinase signaling and activates AKT in acute myelogenous leukemia (AML) cells [48]. Activation of the phosphoinositide-3-kinase/AKT/mTOR pathway causes an mTOR-dependent loss in insulin receptor substrate-1 expression leading to feedback down-regulation of the signaling that occurs through this pathway [49]. Rapamycin inhibits AKT/PKB signaling in cells by decreasing the levels of intact mTORC2 below those needed to maintain the phosphorylation of S473 of AKT/PKB [50]. In the gene microarray analysis in our study, the level of mTOR decreased, and TSC increased. Because P-AKT was decreased (Fig 1B), AKT/TSC/mTOR pathway may also be involved in autophagy induced by heat and shake stress.

Severe or prolonged ER stress induces activation of unique pathways that lead to cell death through apoptosis [51, 52]. Casp12, IRE1/PERK/JNK, and p53 signaling pathways have been directly implicated in ER stress-induced apoptosis [53–55]. The data showed that Casp12 was increased and that the p53 signaling pathway was activated in gene microarray analysis. Especially at the tip of jejunal villi in our study, Casp12 and Casp3 increased (Figs 1B and 3), which may indicate that ER stress-induced apoptosis occurred in response to the stress, suggesting that ER stress may induce autophagy through the AKT/TSC/mTOR pathway, with apoptosis simultaneously mediated by Casp12 or p53 signaling pathways.

In the analysis of the comparison of the network graph of proteins and GO (Fig 6A) and the gene pathway network graph (Fig 6B), AKT1, TSC1/2, Casp9, Fas, Bcl2s, Bcl2ls, Eif-2s, Mmp9, Zbtb16, and others were important. Results of RT-PCR were consistent with the gene microarray analysis and verified the data. In GO cluster analysis, the 2-D view module functional annotation clustering graphic presentations helped predict the possible functions of genes related to ER stress-induced apoptosis and autophagy, and provided new insight into gene function research.

In conclusion, the present study investigated the effects of heat and shake stress on the morphology, differential gene expression, and the level of related proteins in rat jejunum. Stress induced significant morphological damage to the small intestinal epithelium. Fifty-three differentially expressed genes related to apoptosis and autophagy were selected from the gene microarray data. Related proteins such as LC3, Casp12, mTOR, Casp3, and others were also detected. The changes in gene expression provided the potential biological pathways that occur in response to stress. Based on the gene expression analysis, damage to the rat small intestine may be related to apoptosis induced by ER stress at the tip of the jejunal villus, and autophagy also induced by ER stress may be a potential cell survival mechanism in the heat and shake stress response during summer. The AKT/TSC/mTOR pathway may also be involved in this autophagy mechanism. In this study, the relationship between ER stress, apoptosis, and autophagy may provide new perspectives for stress research. Our results also provide a theoretical basis for the mechanisms underlying rat small intestine injury in stress stimulated by simultaneous heat and shake.

## Supporting Information

S1 Dataset Body weight, body temperature(°C) and the level of HSP27 and 70 in Control, S1d and S3d. (XLSX)

S2 Dataset Quantification of western blot determination of P-AKT, AKT, cleaved Casp3 and LC3 in Control, S1d and S3d. (XLSX)

S3 Dataset The RNA expression levels of Zbtb16, Dap, Herpud1, mTOR, Lcn2, Atf6, Casp9, Casp12, Casp8, and Atf4 in Control, S1d and S3d. (XLSX)
